# Prominin 1 is crucial for the early development of photoreceptor outer segments

**DOI:** 10.1038/s41598-024-60989-5

**Published:** 2024-05-07

**Authors:** Sila Yanardag, Scott Rhodes, Thamaraiselvi Saravanan, Tongju Guan, Visvanathan Ramamurthy

**Affiliations:** 1 Department of Biochemistry and Molecular Medicine, West Virginia University, Morgantown, WV USA; 2 Department of Ophthalmology and Visual Sciences, West Virginia University, Morgantown, WV USA

**Keywords:** Retinal diseases, Retina

## Abstract

Prominin 1 (PROM1) is a pentaspan transmembrane glycoprotein localized on the nascent photoreceptor discs. Mutations in PROM1 are linked to various retinal diseases. In this study, we assessed the role of PROM1 in photoreceptor biology and physiology using the PROM1 knockout murine model (rd19). Our study found that PROM1 is essential for vision and photoreceptor development. We found an early reduction in photoreceptor response beginning at post-natal day 12 (P12) before eye opening in the absence of PROM1 with no apparent loss in photoreceptor cells. However, at this stage, we observed an increased glial cell activation, indicative of cell damage. Contrary to our expectations, dark rearing did not mitigate photoreceptor degeneration or vision loss in PROM1 knockout mice. In addition to physiological defects seen in PROM1 knockout mice, ultrastructural analysis revealed malformed outer segments characterized by whorl-like continuous membranes instead of stacked disks. In parallel to the reduced rod response at P12, proteomics revealed a significant reduction in the levels of protocadherin, a known interactor of PROM1, and rod photoreceptor outer segment proteins, including rhodopsin. Overall, our results underscore the indispensable role of PROM1 in photoreceptor development and maintenance of healthy vision.

## Introduction

Photoreceptors are specialized neurons that capture and convert photons into electrical signals^1–3^. These neurons possess elaborate ciliary structures known as outer segments (OS), comprised of tightly stacked discs required to capture photons and initiate phototransduction efficiently^1,4^. The continuous formation of discs occurs through the evagination of a membrane at the base of the OS near the connecting cilium^5,6^, while the phagocytosis of mature discs at the tip of the OS is mediated by the retinal pigmented epithelial (RPE) cells^1,4,7,8^. This dynamic process, where approximately 10% of the mature discs are phagocytosed daily to be replaced by new ones^9–12^, is important for structural integrity and function of photoreceptors. Despite significant advancements in the last few decades, the precise mechanisms underlying the formation and maturation of the discs remain elusive.

Several important clues and insights into photoreceptor development and outer segment morphogenesis have been derived from clinical studies uncovering mutations in genes associated with blindness. One such gene is prominin 1 (*PROM1*), with mutations linked to Retinitis pigmentosa, Stargardt disease, and cone-rod dystrophy^13^. PROM1, located in the nascent photoreceptor discs^14^, is thought to play an important role in disc morphogenesis. Previous studies have suggested that protection from light exposure prevents photoreceptor loss due to deficiencies in PROM1^15^. In this work, we used the retinal degeneration 19 (rd19) mouse model to investigate the need for PROM1 in vision and photoreceptor structure and function. The lack of PROM1 leads to progressive loss of photoreceptor function as early as post-natal day 12 (P12), before eye opening. The functional decline was accompanied by gliosis at P12, preceding cell death that occurs at later ages. Surprisingly, protecting the mice from light exposure did not confer any benefits in terms of photoreceptor function and survival. Interestingly, in rd19 mice, the outer segment discs were malformed, displaying whorl-like membrane structures rather than tightly packed discs. Our findings underscore the critical role of PROM1 in maintaining the structural integrity and function of photoreceptors, providing valuable insights into the development of OS and the pathophysiology of vision disorders linked to PROM1 mutations.

## Results

### Loss of PROM1 leads to a decline in visual function.

**Figure 1:**
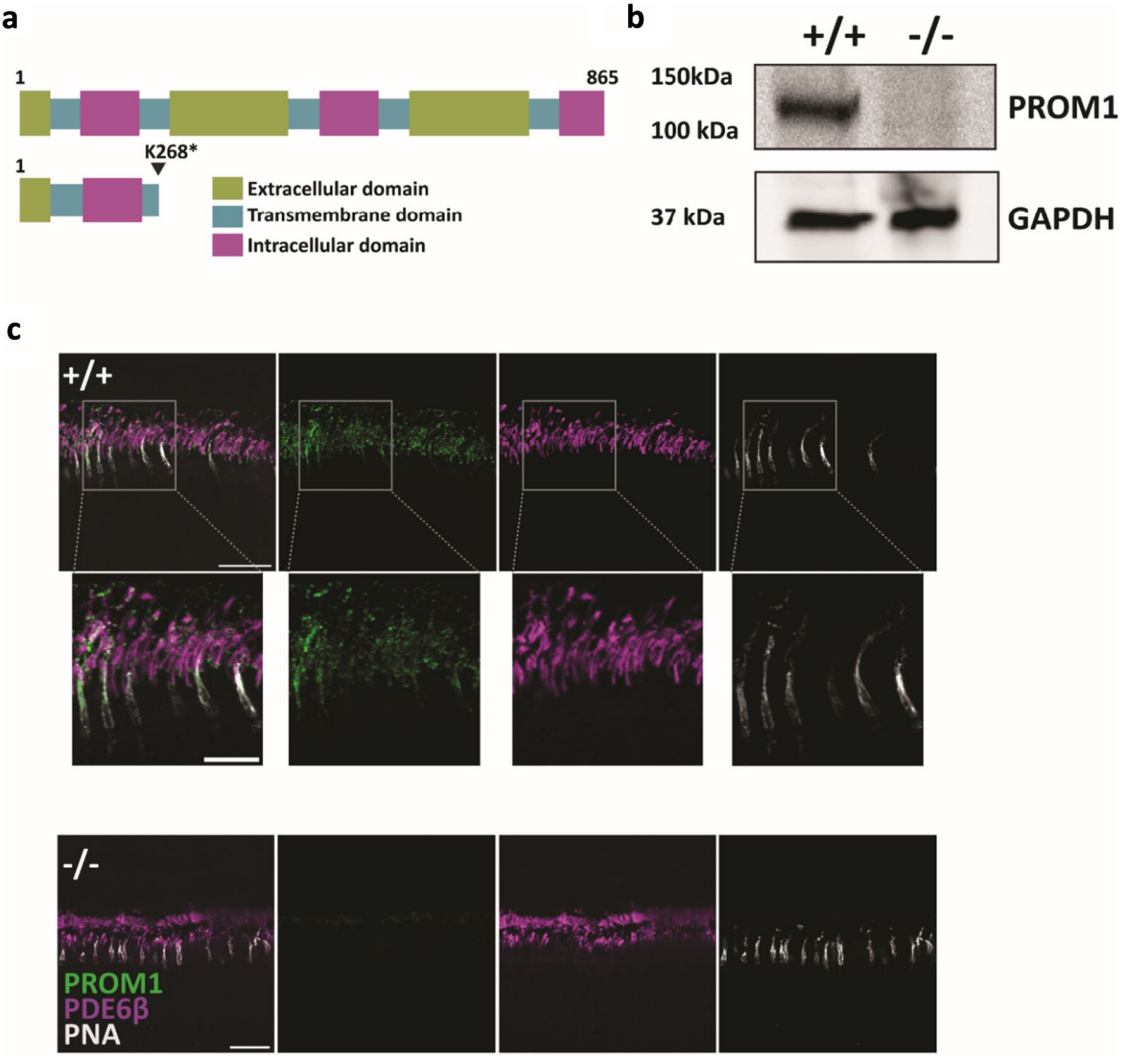
Domain organization and expression of PROM1. (**a**) Schematic showing PROM1, a pentaspan transmembrane protein with three extracellular domains, three intracellular domains, and five transmembrane domains with extracellular N- and intracellular C-terminus. In rd19 mouse model, a nonsense mutation in exon nine in *PROM1* leads to a premature termination at amino acid residue 268 (K268*). (**b**) Immunoblot of retinal lysates isolated from wildtype (+/+) and littermate PROM1 knockout (−/−) mice. Molecular weight markers in kilodaltons (kDa) are indicated on the left. PROM1, detected using monoclonal antibody ab27966, appears above 100 kDa and was absent in retinal lysates from rd19 animals. Housekeeping protein, Glyceraldehyde 3-phosphate dehydrogenase (GAPDH) is used as a loading control. (**c**) Retinal sections from P12 PROM1 knockout (−/−) (bottom) and littermate controls (+/+) (top) stained with indicated markers. PROM1 (mAb13A4) (green) PDE6β (magenta), and PNA (white). Scale bars: 20 µm, inset scale bars: 10 µm.

PROM1 is a ubiquitous pentaspan transmembrane protein (Fig. 1a) with a significantly higher expression in the adult retina^16^. Within the retina, PROM1 is primarily expressed in the base of the rod and cone photoreceptors (Fig. 1c)^14^. To study the importance of PROM1 in photoreceptor function, we used the retinal degeneration 19 (rd19) mouse model. Rd19 was identified as a spontaneous mutation with defects in vision^17^. In the rd19 mouse model, a single nucleotide mutation (A > T) on the PROM1 gene causes a premature stop codon (K268*) (Fig. 1a). We confirmed the absence of PROM1 expression using a monoclonal antibody (ab27699-RRID: AB_940852). In retinal extracts from wildtype animals, we were able to detect a 118 kDa band that corresponds to the expected molecular weight of PROM1 at P12. In contrast, retina lysates from rd19 showed no detectable signal (Fig. 1b, and Supplementary Fig. 1). We also confirmed the lack of PROM1 in rd19 mice using mAB13A4 (RRID: AB_467471) monoclonal antibody via immunohistochemistry (IHC) (Fig. 1c). mAB13A4 recognizes a structural epitope within the third extracellular domain of PROM1^16^. While wildtype retina showed robust PROM1 staining in the outer segments, mostly co-localized with cones and the base of rods (Fig. 1c, insets), the retina from rd19 animals significantly lacked signal for PROM1. Altogether, the immunoblotting and IHC results confirm the absence of PROM1 in the rd19 model, and therefore, from here on, we use PROM1 knockout (−/−) to denote rd19 mice.

**Figure 2:**
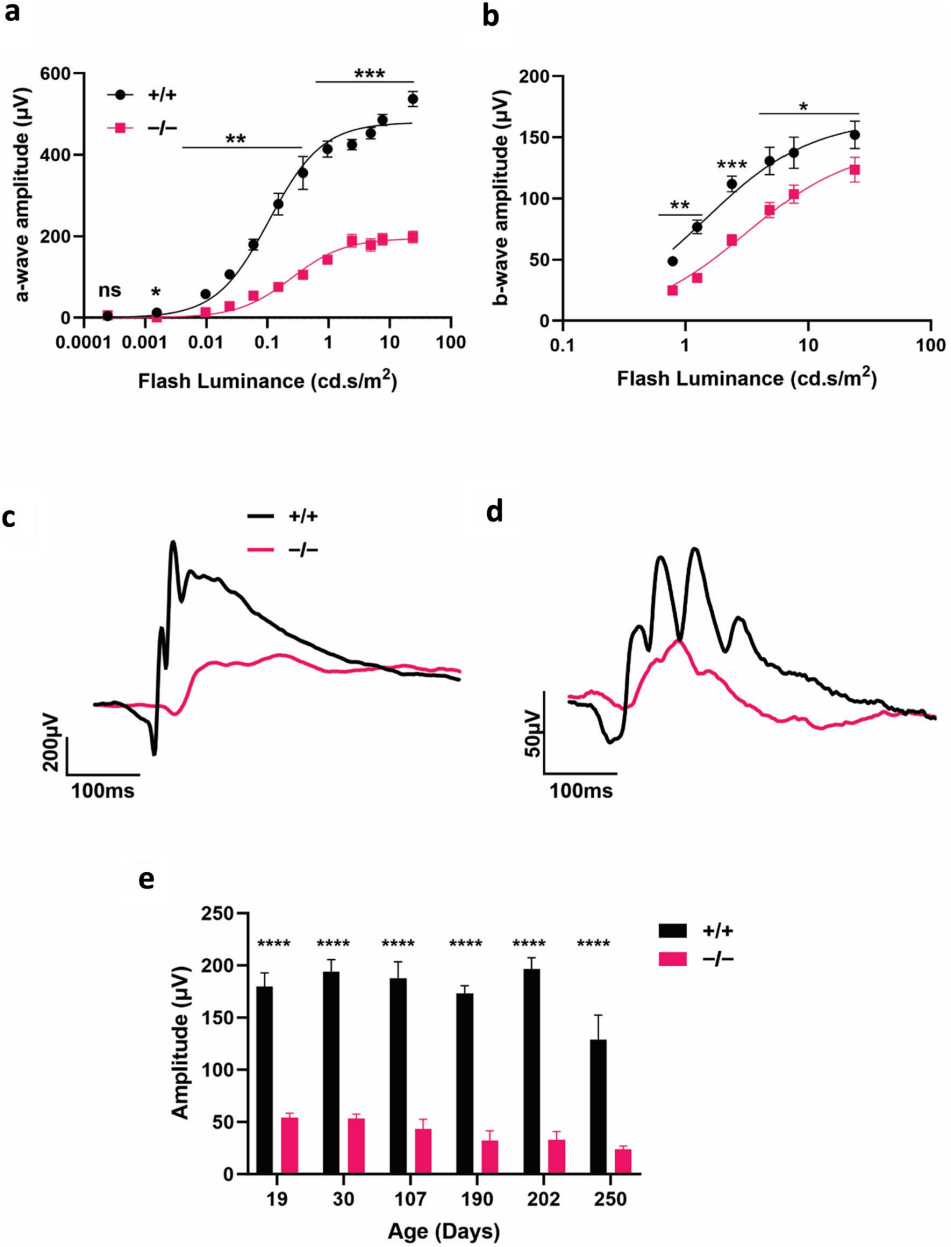
Progressive loss of photoreceptor function in the absence of PROM1. Photoreceptor function of wildtype (+/+) and littermate PROM1 knockout (−/−) mice are tested using electroretinography (ERG). (**a**,**b**) Sensitivity curves show reduced scotopic a-wave (**a**) and photopic b-wave (**b**) response at P19. Curve fit parameters for +/+ and −/− are A_max_ = 480 ± 17 µV, I_½_ = 0.1073 ± 0.025 cd.s/m^2^ and A_max_ = 195.4 ± 8.6 µV, I_½_ = 0.2586 ± 0.0545 cd.s/m^2^, respectively. (**c**,**d**) Representative scotopic response curve at 0.059 cd.s/m^2^ (**c**), and photopic response curve measured at 7.6 cd.s/m^2^ (**d**). (**e**) progressive reduction of rod function, as indicated by the amplitude of scotopic-a wave during the lifetime of PROM1 knockout (−/−) mice. (unpaired t-test, "ns" denotes "not significant" p-value > 0.05, **p-value = 0.0061, ***p-value < 0.0001, n = 3–4).

To investigate the importance of PROM1 in photoreceptor function, we performed electroretinography (ERG). ERG is a non-invasive tool that detects the response of photoreceptors to increasing light stimuli. The negative tending a-wave measures the hyperpolarization of photoreceptors in response to light, and the positive b-wave arises from downstream bipolar cells (see Fig. 2c,d black lines representing ERG traces recorded from wildtype mice). We periodically measured the scotopic (rod) and photopic (cone) responses of the PROM1 knockout animals and littermate controls using ERG. We evaluated visual function from P19, at the time of weaning, up to P250. At P19, the comparison of sensitivity curves shows that both scotopic a-waves and photopic b-waves are significantly reduced in PROM1 knockout animals, implying diminished rod function (Fig. 2a) and cone function (Fig. 2b), respectively. The representative ERG scotopic a-wave (Fig. 2c) and photopic b-wave (Fig. 2d) show vastly reduced ERG responses in animals lacking PROM1. When we compared the progression of rod photoreceptor functional loss, we noted that the rod function of PROM1 knockout animals is significantly reduced compared to their littermate controls at all ages (Fig. 2e). These results show that lack of PROM1 is crucial for the photoreceptor function throughout the lifetime of the mice.

### Light protection does not preserve photoreceptor function

A previous study showed light-dependent exacerbation of photoreceptor loss in the absence of PROM1^15^. In that study, when authors raised the PROM1 knockout mice in the dark, the photoresponse was robust in comparison to the knockout mice reared in normal light. The dark rearing also protected the photoreceptors lacking PROM1 from death^15^. Thus, we wanted to evaluate whether light protection preserves the visual health of PROM1 knockout animals.

Rd19 mice from Jackson lab are in C57Black6/J background. It is known that C57Black6/J carries a hypomorphic *rpe65* allele that codes for methionine at position 450. To rule out the effects of this allele in protection observed by dark-rearing of PROM1 knockout animals, we crossed C57Black6/J with 129SV/E that carries *rpe65* allele coding for leucine at position 450, similar to humans. After a few crosses, we obtained PROM1 knockout animals with RPE65 that carried leucine at position 450.

**Figure 3:**
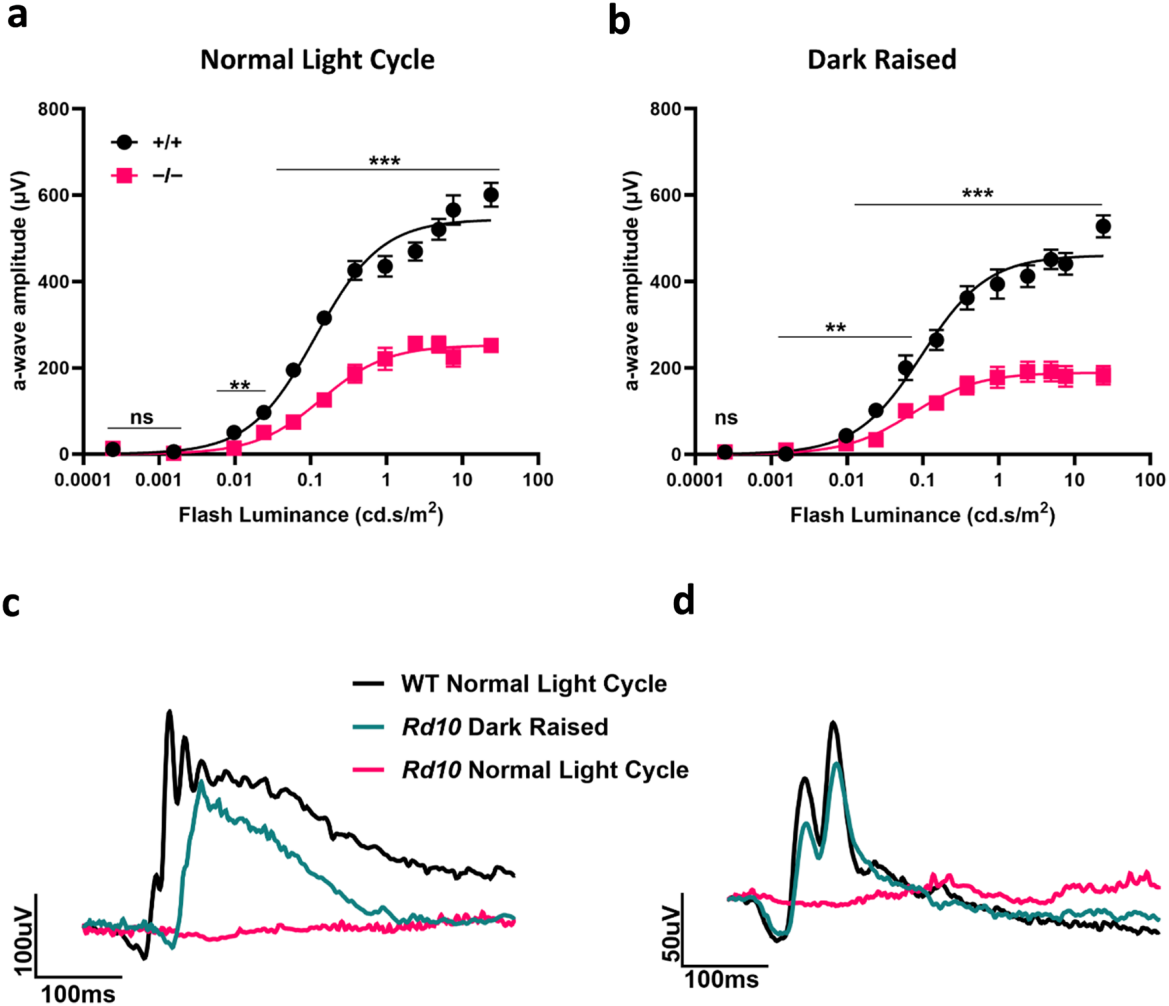
Dark rearing does not preserve photoreceptor function in animals lacking PROM1. (**a**) Sensitivity curve of rod function of normal light raised littermate wildtype (+/+) and PROM1 knockout (−/−) mice at P30. Curve fit parameters are as follows: normal light cycle wildtype A_max_ = 544.1 ± 20 µV, I_½_ = 0.1157 ± 0.02243 cd.s/m^2^, normal light cycle knockout A_max_ = 252.3 ± 9.9 µV, I_½_ = 0.1367 ± 0.0271 cd.s/m^2^ (**b**) Sensitivity curve of rod function of dark raised littermate wildtype (+/+) and PROM1 knockout (−/−) mice at P30. Curve fit parameters are as follows: dark raised wildtype A_max_ = 460.0 ± 16.1 µV, I_½_ = 0.09717 ± 0.01844 cd.s/m^2^, dark raised knockout A_max_ = 189.1 ± 8 µV, I_½_ = 0.07231 ± 0.01609 cd.s/m^2^. (**c**,**d**) rd10 mice were used as positive control. rd10 mice raised in the same experimental conditions from birth to P30. (**c**) Representative scotopic responses measured at 0.159 cd.s/m^2^ (**d**) and photopic responses measured at 7.6 cd.s/m^2^ (unpaired t-test, "ns" denotes "not significant" p-value > 0.05, *p-value = 0.0239, ***p-value < 0.0001, n = 3–4).

We raised PROM1 knockout and littermate controls in complete darkness and normal light cycle in the vivarium for 30 days starting from birth. We compared the photoreceptor function of PROM1 knockout to their littermate controls. Our findings showed dark raising had no significant impact on the preservation of rod photoreceptor function (Fig. 3a,b). In a normal light cycle, knockout animals showed about 46% reduction in scotopic photoreceptor response (Fig. 3a). A similar level of loss (about 41%) in photoreceptor function was noted in dark-raised animals (Fig. 3b).

Given the previous publication that noted background as a possible modifier in protection^15^, we hypothesized that the *rpe65* allele plays a crucial role in this process. Therefore, we investigated if dark raising protected visual function in PROM1 knockout animals in C57Black6/J background with hypomorphic *rpe65* allele methionine at position 450 (RPE65 M/M). Additionally, we conducted a similar experiment with the PROM1-cre-ERT2 model, where cre-ERT2 is expressed instead of PROM1, leading to a loss in PROM1 expression. Our results showed consistently reduced photoreceptor response from PROM1 knockout mice in both PROM1-cre-ERT2 and RPE65 M/M models. The scotopic a-wave amplitudes of PROM1-cre-ERT2 mice in response to a light stimulus of 0.025 cd.s/m^2^ were reduced by 67% (109.9 ± 12.7 +/+ vs. 35.1 ± 6.7 −/−). Similarly, the a-wave amplitudes of PROM1 knockout (RPE65 M/M) mice in response to the same light stimulus were reduced by 42% (57.1 ± 17.1 +/+ vs 33.4 ± 10.3 −/−) (Supplementary Fig. 2). Regardless of the background and the knockout model used in this study, dark raising did not preserve photoreceptor function in PROM1 knockout mice.

To ensure the integrity of our experimental setup, we evaluated our dark-raising conditions using the rd10 animal model. Rd10 animals carry missense mutation in exon thirteen of the *phosphodiesterase-6β* (*PDE6β*) gene, leading to R560C mutation in the encoded protein. These animals undergo photoreceptor cell death and, thus, vision loss when reared in normal light conditions in the vivarium^18,19^. We and other groups previously showed that dark-rearing rd10 animals preserve the photoreceptor function^19–21^. We used the same experimental conditions, similar to those we used for PROM1, where the rd10 breeding pair is kept in the dark until the offspring are 30 days old. When the ERG results of normal light cycle raised rd10 mice were compared to dark raised rd10 mice and wildtype C57Black6/J mice, we found that light protection indeed preserved the visual function of rd10 mice (Fig. 3c,d). These findings validated the experimental conditions we employed. Overall, our findings show that dark rearing fails to preserve photoreceptor function in mouse models that recapitulate the disease state caused by mutations resulting in PROM1 ablation.

### PROM1 is essential for photoreceptor survival, regardless of light exposure

**Figure 4:**
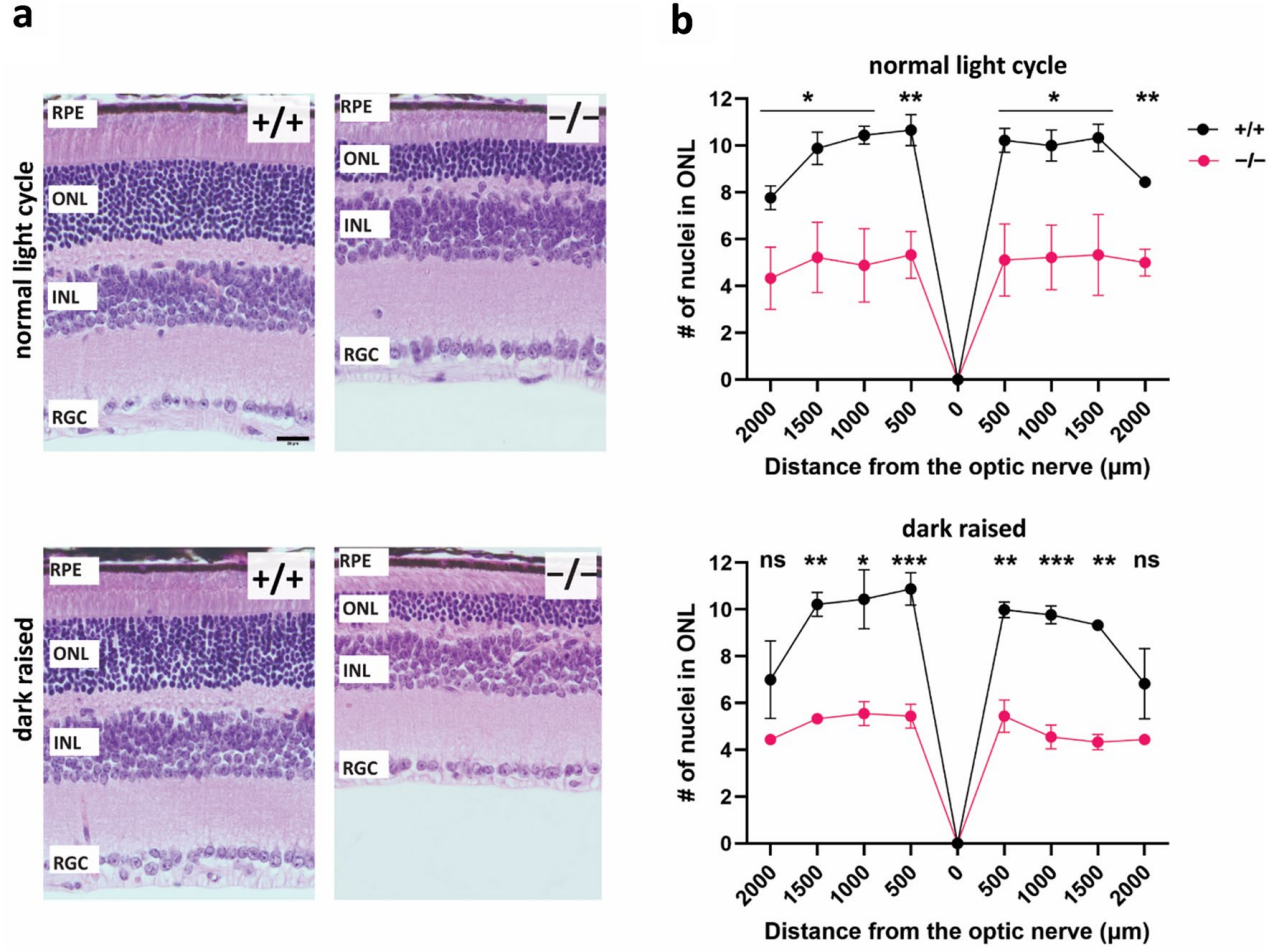
Photoreceptor cell death in PROM1 knockout retina. (**a**) Representative hematoxylin and eosin stained (H&E) histology images of retina from wildtype (+/+) (Top) and PROM1 knockout (−/−) mice (Bottom) at P35 raised in normal light cycle and complete darkness. (**b**) Spider plot analysis of H&E images similar to those from panel (**a**). Starting from the optic nerve, nuclei of the outer nuclear layer (ONL) were counted every 500 µm. (unpaired t-test, "ns" denotes "not significant" p-value > 0.05, **p-value = 0.0019, ***p-value = 0.0008, n = 3 biological replicates and n = 3 technical replicates). *RPE*: *Retinal Pigmented Epithelium,* *ONL:* *Outer Nuclear Layer*, *INL:* *Inner Nuclear Layer*, *RGC*: *Retinal Ganglion Cells*. Scale Bar: 20 µm.

Next, we sought to identify whether reduced photoreceptor function is accompanied by photoreceptor degeneration in PROM1 knockout mice. To answer this question, we stained retinal cross-sections of P35 mice with hematoxylin and eosin and imaged them by light microscopy (Fig. 4a). The thickness of the retina's outer nuclear layer (ONL) is used to measure retina health indirectly. When the retina of the PROM1 knockout mice was compared to their wildtype littermate controls raised in a normal light cycle, we found that the number of nuclei in the ONL was reduced by about twofold (Fig. 4b). A similar observation was also noted in mice raised in complete darkness (Fig. 4b), indicating that light protection does not prevent photoreceptor death in PROM1 knockout mice. The results of the histological analysis of the tissue align with our ERG data (Fig. 3a,b), which also show no improvement in the photoreceptor function of PROM1 knockout mice when they were protected from light exposure. Overall, these data show us that light protection cannot compensate for the outcomes caused by the loss of PROM1 in the rd19 mouse model.

### Photoreceptor function is reduced in PROM1 knockout mice before the eye-opening

**Figure 5:**
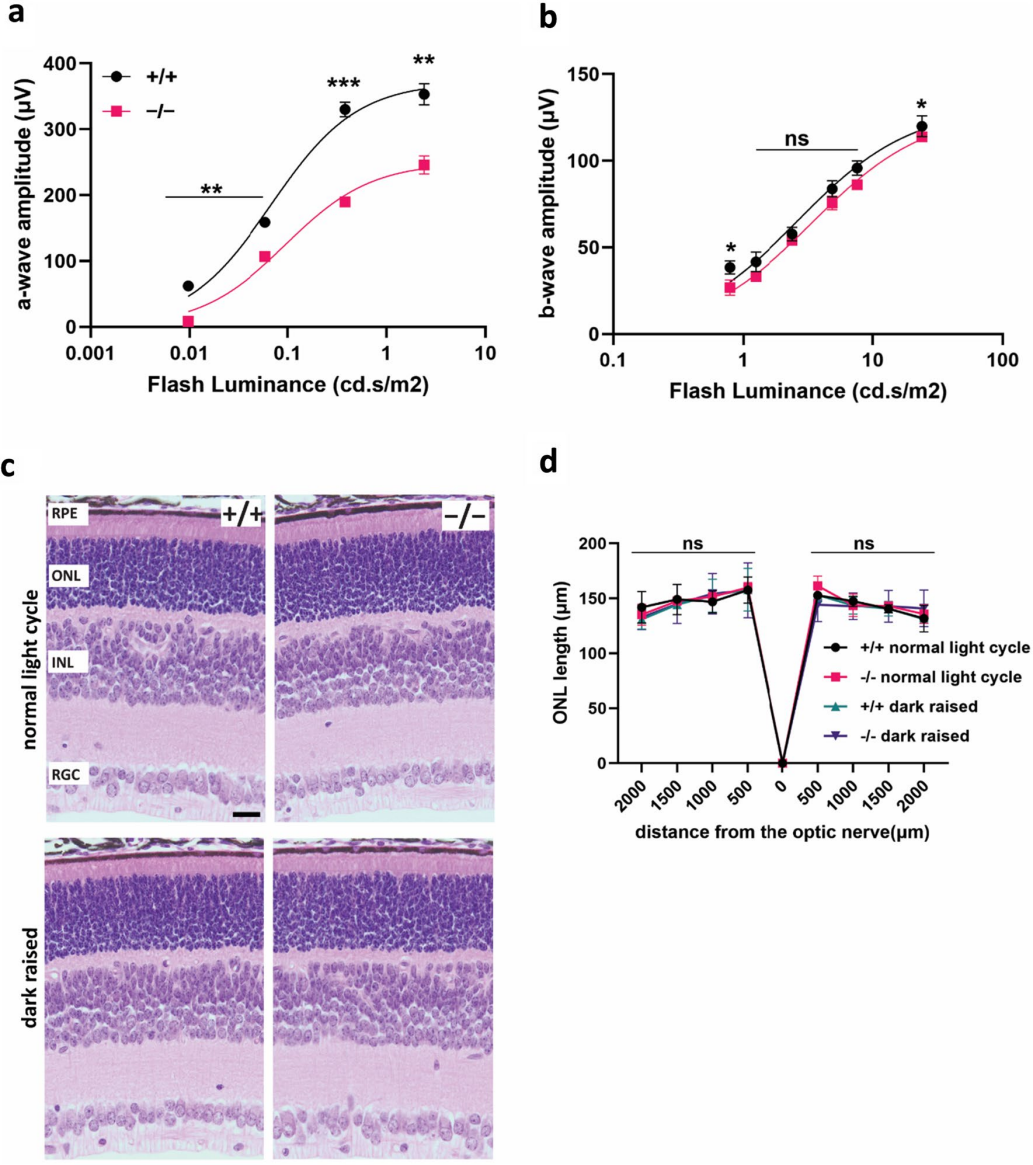
Early reduction in photoreceptor function in PROM1 knockout mice. Photoreceptor function of wildtype (+/+) and PROM1 knockout (−/−) mice tested using ERG. (**a**,**b**) Sensitivity curves show reduced scotopic-a wave (**a**) and photopic-b wave (**b**) response at P12. Curve fit parameters are as follows: wildtype A_max_ = 371.7 ± 21.1 µV, I_½_ = 0.06814 ± 0.01593 cd.s/m^2^, PROM1 knockout A_max_ = 249.8 ± 17.5 µV, I_½_ = 0.09501 ± 0.02582 cd.s/m^2^ (unpaired t-test, "ns" denotes "not significant" p-value > 0.05, *p-value = 0.0101, ****p-value < 0.0001). (**c**) Representative hematoxylin and eosin (H&E) stained histology images of retina from wildtype (+/+) and PROM1 knockout (−/−) mice raised in the normal light cycle and complete darkness. (**d**) Spider plot analysis of H&E images in panel C. Starting from the optic nerve, nuclei of the outer nuclear layer (ONL) were counted every 500 µm (unpaired t-test, "ns" denotes "not significant" p-value > 0.05, n = 3 biological replicates and n = 3 technical replicates). *RPE: Retinal Pigmented Epithelium, ONL: Outer Nuclear Layer, INL: Inner Nuclear Layer, RGC: Retinal Ganglion Cells*. Scale Bar: 20 µm.

PROM1 is a stem cell marker expressed in the hematopoietic and neuroepithelial stem cells. It plays a role in cell fate decision, stem cell maintenance, and expansion^22,23^. The involvement of PROM1 in embryonic development prompted us to examine whether PROM1 is needed for photoreceptor development. Our functional analysis by ERG shows that at P12, before the mice opened their eyes, photoreceptors of PROM1 knockout mice had reduced scotopic response to the light stimuli compared to the wildtype mice. However, the photopic ERG response is not significantly altered (Fig. 5a,b). The retinas collected from PROM1 knockout mice did not display any gross abnormalities by light microscopy, such as reduced ONL length indicative of impaired photoreceptor cell development or death. Light protection had no impact on the retinal structure and photoreceptor development (Fig. 5c). Apart from qualitative observations, quantitative analysis of ONL length yielded no significant differences between wildtype and PROM1 knockout mice at P12 (Fig. 5d).

### The retina of PROM1 knockout mice has a higher number of activated microglia

**Figure 6:**
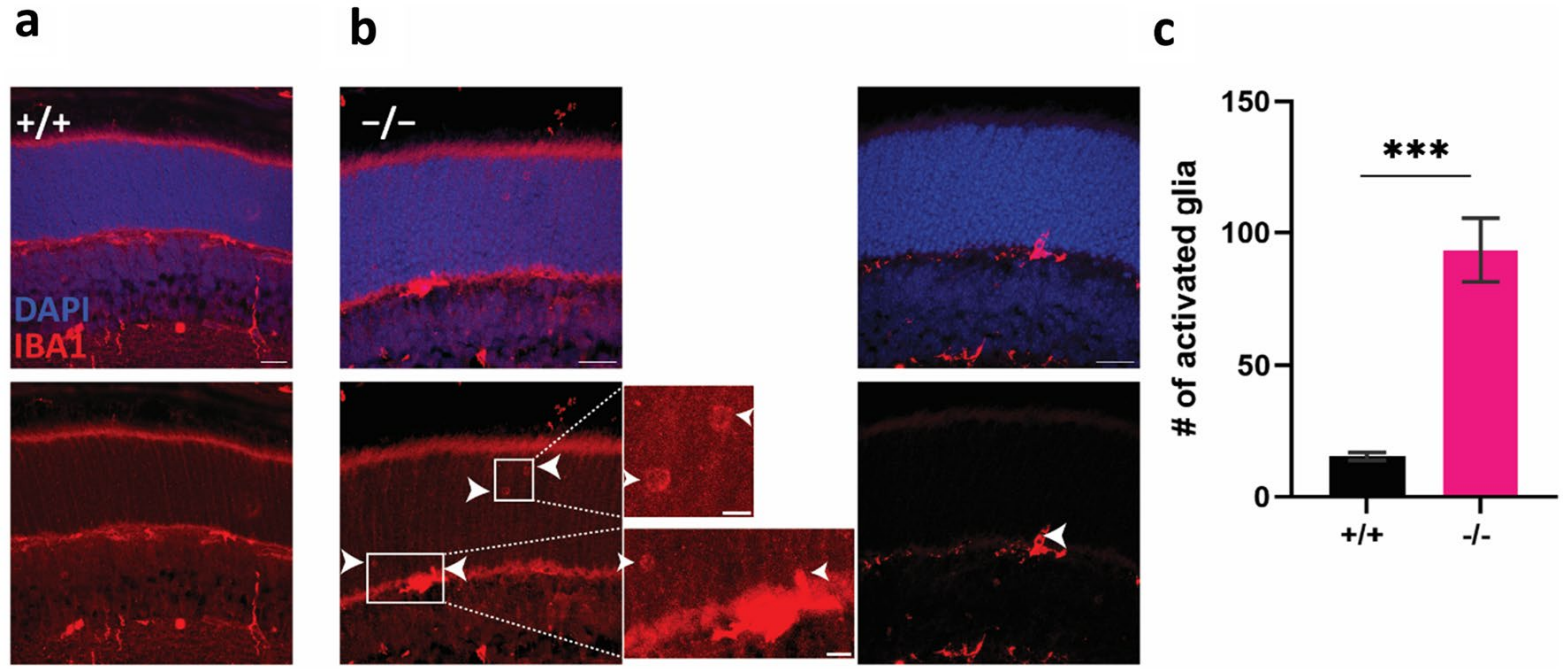
Lack of PROM1 triggers early gliosis. Representative immunohistochemistry images show wildtype (+/+) (**a**) and littermate PROM1 knockout (−/−) (**b**). Retinal sections are stained with DAPI (blue) marking nuclei and IBA1 (red), a microglial marker. Arrowheads point at activated microglia in the outer nuclear layer. Insets show the IBA1 staining in the outer nuclear layer (ONL). (unpaired t-test, ***p-value=0.0004). Scale bars: 20 µm, inset scale bars: 5 µm.

Although the rod function of PROM1 knockout mice was reduced, the photoreceptor health seemed unaffected. Thus, we sought to determine if there was any glial response indicative of photoreceptor damage. We stained the retinal sections from wildtype and PROM1 knockout mice at P12 with ionized calcium-binding adaptor molecule 1 (IBA1), a marker for microglia activation, to determine whether PROM1 knockout retina has higher amounts of activated microglia sensing early photoreceptor damage. Results showed increased activated microglia in the retina isolated from PROM1 knockout mice (Fig. 6b, white arrowheads) compared to the littermate controls (Fig. 6a–c). The magnified images depict the IBA1 staining in the photoreceptor outer nuclear layer (Fig. 6b, insets). These findings serve as early indicators of declining photoreceptor health preceding cell death.

### The outer segment of PROM1 knockout mice displays disc dysmorphogenesis

**Figure 7:**
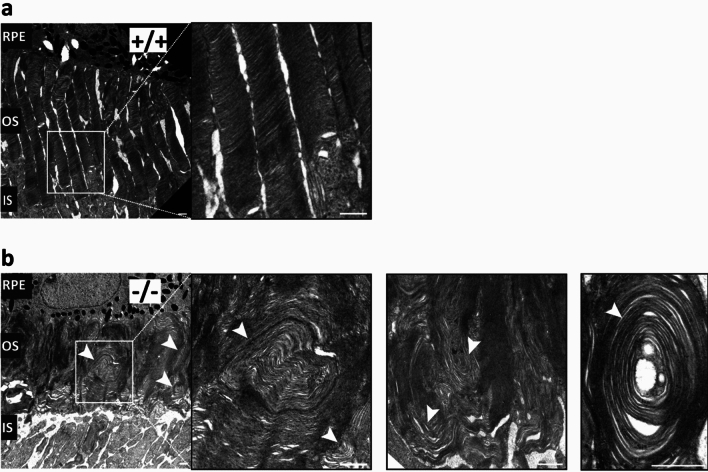
PROM1 is essential for the architectural integrity of photoreceptor outer segments. (**a**) Electron microscopy images of wildtype (+/+) retinal sections show properly organized and tightly stacked outer segment discs. (**b**) Electron microscopy images of PROM1 knockout (−/−) retinal sections show disc dysmorphogenesis with round extended membrane structures (white arrowheads) instead of stacked discs. *RPE: Retinal Pigmented Epithelium, OS: Outer Segment, IS: Inner Segment.* Scale bars: 1 µm.

Reduced rod function and morphologically unaltered retina in PROM1 knockout mice at P12 (Fig. 5) indicate defects in photoreceptor ultrastructure. Since PROM1 is prominently localized on the membrane protrusions^24^, we hypothesized that PROM1 is essential for the photoreceptor outer segment architecture, specifically disc formation and morphogenesis. We used transmission electron microscopy (TEM) imaging to test this hypothesis and compared the photoreceptor outer segments of PROM1 knockout mice to the littermate controls. We found that PROM1 knockout mice do not develop structurally sound outer segments (OS). In PROM1 knockout mice, the laterally stacked discs (Fig. 7a) were replaced by whorl-like continuous membrane extensions (Fig. 7b), pervasive throughout the retina. We conclude that the malformed photoreceptor outer segments were the reason for the reduced photoreceptor function of PROM1 knockout mice starting from P12, before eye opening.

### Phototransduction proteins are reduced in PROM1 knockout mice

**Figure 8:**
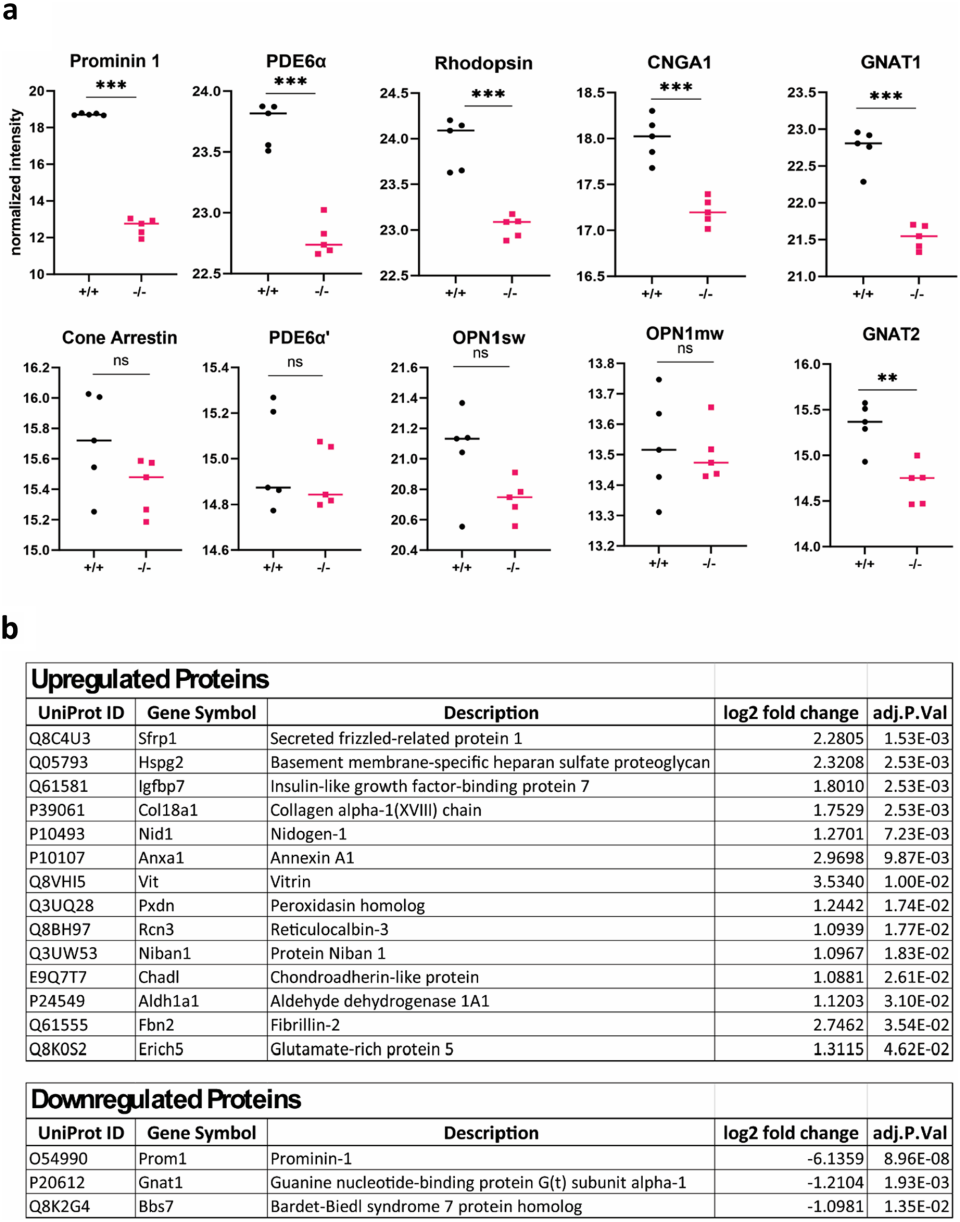
Loss of PROM1 causes a reduction in OS resident proteins. (**a**) Proteomics analysis of the PROM1 knockout (−/−) retina at P12 shows a significant reduction in rod proteins (top), while cone proteins (bottom) are mostly unchanged. (**b**) Full list of differentially regulated proteins in wildtype and PROM1 knockout retina. (Unpaired t-test, "ns" denotes not significant p-value > 0.05, **p-value = 0.0027, ***p-value < 0.0001).

Next, we asked whether the loss of PROM1 affected the protein composition in the photoreceptors. To obtain a comprehensive and unbiased answer to this question, we utilized tandem mass tag (TMT) proteomics to compare the retinas of PROM1 knockout mice to those of wildtype mice at P12. With this approach, we identified 6,069 proteins in our samples. Out of 6,069 proteins, 17 were found to be differentially regulated at (Fig. 8b) (log2 fold change = 1 and p-value ≤ 0.05). When the fold change criteria were excluded from the analysis, we found 170 differentially regulated proteins. A complete list of differentially regulated proteins can be found in Supplementary Table 1.

TMT-based proteomics results confirmed the depletion of PROM1 (adj. p-value: 8.96 × 10^–8^). Additionally, we found a significant reduction in rod phototransduction proteins such as rhodopsin, transducin, phosphodiesterase 6 (PDE6), and cyclic nucleotide gate channel subunits (CNG) (Fig. 8a). Interestingly, in agreement with the functional analysis, cone outer segment proteins did not show significant changes in the absence of PROM1 at P12 (Fig. 8b). We observed a reduction of protocadherin (CDHR), retina-specific phospholipid-transporting ATPase (ABCA4), peripherin 2 (PRPH2), and rod outer segment membrane protein 1 (ROM1) (Supplementary Table 1). These results show that lack of PROM1 causes structural abnormalities, resulting in the loss of outer segment proteins essential for the structure and function of photoreceptors.

## Discussion

The central observation in this work is that prominin 1 (PROM1) is essential for normal vision and the development of outer segment discs. The absence of PROM1 in the rd19 murine model leads to significant defects in outer segment morphology and loss of photoreceptor function at P12 before the eye opening, which deteriorates as the mice age. The unbiased proteomics demonstrates that a reduction in the levels of OS-resident proteins accompanies the defective outer segment (OS) morphology. These findings align with the clinical observations and previously published studies^15,25–27^. In keeping with our observation that OS defects occur before eye opening, dark rearing did not alter the vision loss observed in rd19 and PROM1-cre-ERT2 animals. Our study represents a comprehensive investigation into the role of PROM1 in vision and retinal health across multiple genetic backgrounds. We found that PROM1-mediated blindness is light-independent and manifests at a very young age, prior to eye-opening, thus emphasizing PROM1's exclusive role in photoreceptors as a protein important for the normal development of outer segments.

We observed an early loss of rod function, followed by deficiencies in cones (Fig. 5a,b). However, despite the functional deficits, there was no apparent loss of photoreceptors at P12 (Fig. 5). Interestingly, we noted increased glial activation and migration of microglia in these animals, indicating diminishing photoreceptor health (Fig. 6). The activation of glia was supported by increased expression of Annexin A1, a marker for microglial activation^28^ (Fig. 8b). By four weeks, the photoreceptor nuclear layer was reduced by half (Fig. 4), indicating the necessity for PROM1 in the survival of photoreceptor cells.

In a previous study, vision loss and photoreceptor cell death due to PROM1 deficiency were compensated by protecting the animals from light exposure^15^. PROM1 knockout animals kept in the darkness for 30 days starting from birth showed significantly improved visual responses. Additionally, photoreceptors of PROM1 deficient mice did not undergo cell death when the mice were not exposed to light. The authors observed a strain-specific difference in the protection afforded by dark rearing^15^. In this study, we investigated whether the vision loss observed in the rd19 animal model could be mitigated by dark rearing. Both photoreceptor function (Fig. 3) and survival (Fig. 4) were affected, irrespective of lighting. We hypothesized that commonly observed hypomorphism of the *rpe65* allele^29^, a change in amino acid residue at position 450, might contribute to observed strain differences in the protection afforded in dark rearing. Our observation revealed no protection, regardless of the *rpe65* allele variant. We also evaluated light protection in PROM1 animal models available from Jackson Labs, where cre-ERT2 is expressed instead of PROM1, effectively creating a PROM1 knockout (Supplementary Fig. 1). Overall, across all animal models we tested, PROM1 depletion leads to a loss of photoreceptor function and cell death regardless of rearing conditions.

Since we observed an early functional loss with no significant changes in photoreceptor cell numbers, we sought to determine whether the lack of PROM1 causes any structural defect in the retina. Unlike regularly stacked outer segment discs in wildtype controls, we observed whorl-like contiguous membranes in the PROM1 knockout mice (Fig. 7b). These findings are similar to the observations in two independent animal models, xenopus and mouse^26,27,30^. The mechanisms behind the need for PROM1 in the maintenance or development of OS structure are still not known. Previous studies suggested the loss of PROM1 interacting partner Protocadherin 21 (CHDR), a cadherin homolog in the retina, as a potential mechanism for defective OS development^26,30,31^. In Xenopus laevis, depletion of CHDR phenocopies PROM1 null animals. The authors observed abnormal orientation of discs and defects in outer segments; however, they noted that photoreceptor function was not significantly affected. Additionally, double knockout animals (PROM1/CHDR null) showed no further reduction in ERG responses compared to PROM1 null animals^26^. A transgenic murine model expressing human PROM1 carrying patient mutation R373C showed similar outer segment defects, where the disc membranes are overgrown and abnormally oriented. Furthermore, the authors found that exogenous PROM1 R373C and endogenous CHDR were mislocalized in the myoid region instead of their normal localization in the nascent discs. Pull-down assays using a cell culture system confirmed the interaction of PROM1 with CHDR and actin^30,32^. Notably, our unbiased TMT-based proteomics data showed that the relative abundance of CHDR is significantly reduced in PROM1 knockout mice (adj p-value: 0.0015) (Supplementary Table 1) at P12 before eye-opening, suggesting a dependence of CDHR abundance on PROM1. Further studies are needed to untangle the role of PROM1 interaction with cadherins and actin cytoskeletal elements in the development of the outer segment.

Mutations in PROM1 cause vision phenotype despite its widespread expression throughout the body. Most of the patient mutations of PROM1 are autosomal recessive mutations causing premature stop codon, which is recapitulated in the rd19 murine model used in this study. In contrast, missense mutations are mostly found to be autosomal dominant, leading to Stargardt disease. One possible explanation for why mutations in PROM1 are linked to severe vision phenotypes is the lack of prominin 2 (PROM2) expression in the retina^32^. PROM2 is the structural homolog of PROM1 with 26% and 29% sequence identity in humans and mice, respectively^32^. Like PROM1, PROM2 also localizes on the membrane protrusions. While PROM1 is enriched in the retina (264.6 normalized transcripts per million (nTPM) in the retina vs 52.6 nTPM in the second highest expressing tissue, the salivary gland), PROM2 is enriched in the esophagus with considerably low expression levels in the retina (85.6 nTPM in the esophagus vs 0.4 nTPM in the retina)^33^. The presence of PROM2 in tissues other than the retina might play a compensatory role in the cases of PROM1 mutations, which exclusively result in vision loss.

PROM1 expression was observed in both photoreceptor and retinal pigmented epithelium (RPE). Consequently, the functional deficits we observed could potentially stem from deficiencies in RPE. Previous studies have suggested that vision loss in PROM1-deficient animals could be due to defective phagocytosis by RPE cells. The murine model used in this study is a whole-body depletion of PROM1, making it unsuitable for distinguishing the role of PROM1 in different cell types. We conclude that the results observed in this study are attributable to photoreceptor defects, as we observed structural and functional defects in photoreceptors at a very young age. Additionally, the mechanism by which photoreceptors degenerate without PROM1 remains unclear. Further investigation is necessary to address this critical issue by using conditional knockouts or the re-introduction of PROM1 in the retina under the cell-type specific promoter.

## Methods

### Ethics statement

All experimental procedures involving animals in this study are performed according to the ARRIVE guidelines and approved and conducted per guidelines stated by the Institutional Animal Care and Use Committee of West Virginia University and the Association for Research in Vision and Ophthalmology.

### Animal care and maintenance

The *Prom1* rd19 mice were acquired from the Jackson laboratory (B6. BXD83-Prom1rd19/Boc, RRID:IMSR_JAX:026803). These rd19 animals are in C57Black/6J background and carry a hypomorphic RPE65 mutation with methionine at position 450 (Rd19 M/M). To generate a mouse line with the RPE65 allele coding for leucine at position 450, we crossed the rd19 mice with 129SVE/J mice. This line, referred to as PROM1 knockout, is used throughout this study unless specified otherwise and is maintained in a mixed background. The mice were housed in the West Virginia University animal facility with a 12-h light/dark cycle with ad libitum access to food and water.

For dark rearing, mating pairs were raised in complete darkness, without any light. In dark rearing rooms, mice were placed in a temperature- and humidity-controlled incubator (Powers Scientific Inc., cat# HIS33SD). The glass doors of the incubators were covered with light-insulating material to ensure complete darkness. Temperature and humidity were monitored daily by the lab personnel, and red light was used for maintenance tasks. *Prom1* Cre-ERT2 mice (B6N;129S-Prom1tm1(cre/ERT2)Gilb/J, RRID:IMSR_JAX:017743) and rd10 mice (B6.CXB1-Pde6brd10/J, RRID:IMSR_JAX:004297) were obtained from Jackson Laboratories.

### Genotyping

Genotyping was performed using genomic DNA extracted from ear punch samples. Primers used for genotyping and PCR conditions are listed in Supplementary Table 3.

### Immunoblotting

Mice were euthanized using 3% CO_2_ inhalation following the guidelines in the IACUC-approved protocol. After enucleation of the eyes, the retina was collected and kept frozen at – 80 °C until use. Retinas were lysed in RIPA buffer (150 mM NaCl, 1% NP40, 0.5% DOC, 0.1% SDS, 50 mM Tris, final pH 7). 20–40 µg of total protein, measured using a BCA assay kit (Thermo Fisher Scientific, cat# 23225) in 1× SDS protein loading buffer (Boster Bio, cat# AR1112), was loaded in a precast SDS-PAGE gel (GenScript, Cat# M00656). After the SDS-PAGE run and protein separation, gel content was transferred to the PVDF membrane. We also quantified the total protein transferred to the membranes using the Li-COR total protein quantification kit (Li-COR Biosciences, cat# 926-11015). The membrane was blocked with protein-free blocking buffer (Thermo Scientific cat# 37570) for 30 min at room temperature. Following the blocking step, membrane was incubated overnight at 4 °C with primary antibodies in a 1:1 mix of blocking buffer and 1 × phosphate buffered saline (137 mM NaCl, 2.7 mM KCl, 10 mM Na_2_HPO_4_, 1.8 mM KH_2_PO_4_) with 0.05% tween 20 (PBS-T). Following the primary antibody incubation, the membrane is washed with PBST thrice for 5 min. Next, the membrane is incubated with fluorescently conjugated appropriate secondary antibodies (1:50,000 in PBS-T) for 2 h at room temperature. Images of the immunoblot are taken with an Amersham Typhoon imager (Cytiva Life Sciences, cat# 29238583). The antibodies used in this study are listed in Supplementary Table 2.

### Histochemistry and immunohistochemistry

Mice were euthanized using 3% CO_2_ inhalation and cervical dislocation as a secondary euthanasia technique. For histological analysis, eyes were enucleated and fixed in a proprietary fixative provided by Excalibur Pathology Inc. (Norman, OK). Hematoxylin and Eosin (H&E) staining was performed at Excalibur Pathology Inc. The slides were imaged with Olympus MVX10 Slide Scanner at 40 × magnification. For immunohistochemistry, eyes were enucleated, and a small incision is made on the cornea. Eyes were fixed in 4% paraformaldehyde (PFA) (Electron Microscopy Sciences cat# 15710) for 30 min at room temperature. Cornea and lens are removed and eye cups are further fixed for 1.5 h in 4% PFA. Following the fixation step, eyes are immersed in 30% sucrose overnight. Next day, eye cups were embedded in optimal cutting temperature (OCT) compound and flash frozen. Frozen eye cups were sectioned using Leica CM1850 cryostat, at 16 µm thickness. Sectioned tissues were blocked with the blocking buffer (10% Goat sera, 0.5% Triton X-100, 0.005% Sodium Azide in 1 × phosphate buffered saline) for 1 h at room temperature. Following the blocking step, tissue sections were incubated overnight at 4 °C with primary antibodies in antibody dilution buffer (5% Goat sera, 1% Triton X-100, 0.005% sodium azide in 1 × phosphate buffered saline), washed 3 × for 10 min with PBS-T, and incubated for 2 h at room temperature with appropriate fluorescently conjugated secondary antibodies. For nuclei staining, tissue is incubated with 4′,6-diamidino-2-phenylindole (DAPI) (1:10,000, Thermo Scientific, cat# 62248) for 5 min. Tissue was then mounted with Prolong gold anti-fade mounting media (Invitrogen, cat# P36934) and covered with a thin cover glass. Images were taken with Nikon Eclipse Ti2 inverted confocal microscope (Nikon Instruments Inc.) and processed with NIH Image J software. The antibodies used in this study are listed in Supplementary Table 2.

### Electroretinography

Electroretinography (ERG) is performed as described previously^18,20^. Briefly; dark adapted mice were anesthetized with 1.5% isoflurane mixed with 2 L/min oxygen. 8% tropicamide and 1.5% phenylephrine hydrochloride mix (1:1) was used to dilate the pupils. Hydroxypropyl methylcellulose (Novartis Pharmaceuticals) was added to the cornea to facilitate electrode contact. Following the placement of reference electrodes, the response from each eye is measured using UTAS BigShot (LKC Technologies). Scotopic responses were measured in complete darkness using white light flashes ranging 2.45 × 10^−4^–2.4 cd.s/m^2^. Following the scotopic recording, mice were light-adapted for 10 min using white light of 30 cd.s/m^2^ amplitude to saturate rod photoreceptors and photopic response was recorded to measure cone photoreceptor response. The curve fit was done using the Michaelis–Menten equation, and statistical analyses were performed using GraphPad Prism software.

### Ultrastructural analyses of photoreceptors

Retinal samples were prepared and imaged using previously published procedures^18,34,35^. Briefly, the enucleated eyes were fixed with 2% paraformaldehyde, 2.5% glutaraldehyde in 100 mM cacodylate, pH 7.4. Using a 20 G needle, a small incision is made in the edge of the cornea. The eyes are incubated in a glass vial containing the fixative for 30–60 min at room temperature. Then, the eyes were transferred to a petri dish containing a drop of 7% sucrose in 200 mM Cacodylate buffer (pH 7.4). After removing the cornea and lens, the eyecups were returned to a glass vial containing the fixative for two days. After fixation, the eyecup was cut into smaller trapezoid pieces and incubated with 2% osmium tetroxide in 0.1 M cacodylate buffer and then with 1% uranyl acetate. Fixed tissue was dehydrated in a graded ethanol series and then embedded into Polybed 812 (Polysciences, Inc.). After mounting the thin sections on a grid, the samples were post-stained with 3% Reynold's lead citrate, and the stained sections were imaged in a JEOLl1010 transmission electron microscope at 80 kV.

### TMT-proteomics

We used retinal tissues from PROM1 −/− mice and wildtype littermates (n = 5). Retinas were collected and frozen immediately in dry ice. Frozen retinas were sent to IDEA National Resources for Quantitative Proteomics (Little Rock, AR). Briefly, proteins extracted from the retina after lysis in RIPA buffer were subjected to trypsin digestion and Tandem mass tag (TMT) labeling (TMT10-plex Isobaric Labeling Kit, Thermo Fisher, Catalog number: 90110). Tandem labeling aided in the concurrent identification and quantitation of peptides. The fragmented peptides were subsequently identified and quantified using a database search, and bioinformatics analysis was performed with Scaffold 4 4.11.1. The data enrichment and profiling were analyzed using Metascape software.

### Statistics

All data are presented as mean ± standard error margin (n ≥ 3, biological replicates). ERG data were analyzed by unpaired t-test. ONL data were analyzed using ANOVA or an unpaired t-test.

### Supplementary Information

Supplementary Figures.

Supplementary Table 1.

Supplementary Table 2.

Supplementary Table 3.

## Data Availability

All the data supporting our findings are contained within the manuscript.
