# Supplementary material for: Prominin 1 is crucial for the early development of photoreceptor outer segments
Source: Sci Rep. 2024 May 7;14:10498. doi: 10.1038/s41598-024-60989-5 (PMC11076519; doi:10.1038/s41598-024-60989-5)
Supplement: Supplementary file 1 — Supplementary Figures. [file 41598_2024_60989_MOESM1_ESM.docx]

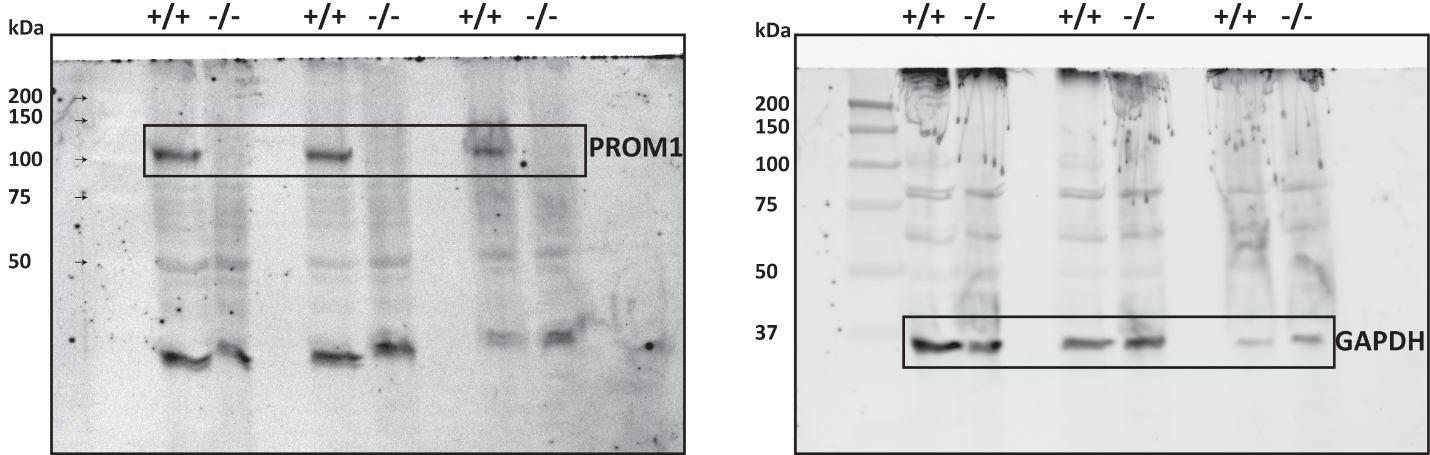


**Supplementary Figure 1**: **Uncropped western blot showing PROM1 depletion in retinas isolated from PROM1 knockout mice at P12.** The blot is probed with antibodies against PROM1 antibody (**left**) and GAPDH (**right**). GAPDH serves as loading control. n=3 mice.


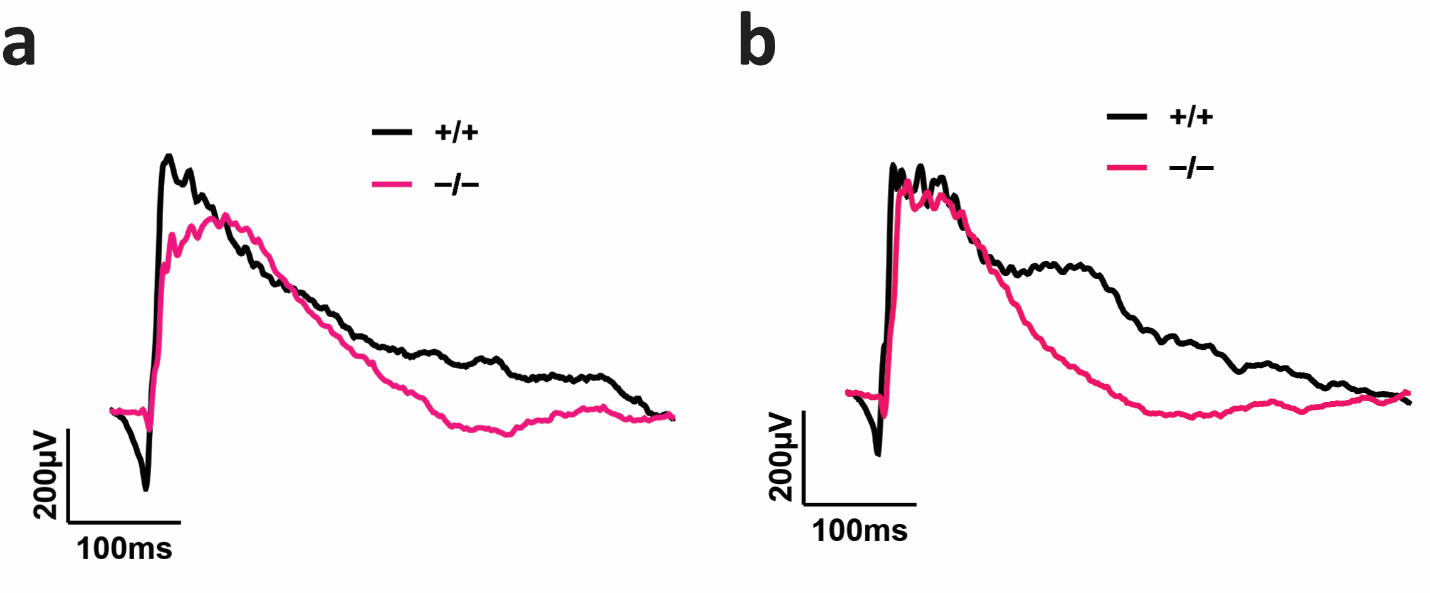


**Supplementary Figure 2**: **Dark rearing does not preserve photoreceptor function in animals lacking PROM1, regardless of the background. (a)** Representative scotopic response of Rd19 RPE65 M/M wild-type (+/+) and PROM1 knockout (-/-) mice at 0.025 cd.s/m2. **(b)** Representative scotopic response of wild-type (+/+)  and cre-ERT2 PROM1 knockout (-/-) mice at 0.025 cd.s/m2.
